# Complete genome sequence of *Peptoclostridium difficile* strain Z31

**DOI:** 10.1186/s13099-016-0095-3

**Published:** 2016-04-01

**Authors:** Felipe L. Pereira, Carlos A. Oliveira Júnior, Rodrigo O. S. Silva, Fernanda A. Dorella, Alex F. Carvalho, Gabriel M. F. Almeida, Carlos A. G. Leal, Francisco C. F. Lobato, Henrique C. P. Figueiredo

**Affiliations:** 1 National Reference Laboratory for Aquatic Animal Diseases (AQUACEN), Ministry of Fisheries and Aquaculture, Federal University of Minas Gerais, Belo Horizonte, Brazil; 2 Veterinary School, Federal University of Minas Gerais, Belo Horizonte, Brazil; 3 Department of Preventive Veterinary Medicine, School of Veterinary, Federal University of Minas Gerais, Av. Antônio Carlos 6627, Pampulha, 30161-970 Belo Horizonte, MG Brazil

**Keywords:** *Peptoclostridium* (*Clostridium*) *difficile*, Live vaccine, Genome sequencing, Competitive exclusion

## Abstract

**Background:**

*Peptoclostridium* (*Clostridium*) *difficile* is a spore-forming bacterium responsible for nosocomial infections in humans. It is recognized as an important agent of diarrhea and colitis in several animal species and a possible zoonotic agent. Despite the known importance of *P. difficile* infection in humans and animals, no vaccine or other effective measure to control the disease is commercially available. A possible alternative treatment for *P. difficile* infection is the use of a nontoxigenic strain of *P. difficile* as a competitive exclusion agent. However, a thorough knowledge of this strain is necessary for this purpose. We selected *P. difficile* Z31, a nontoxigenic strain (PCR ribotype 009), for investigation because it prevents *P. difficile* infection in a hamster model.

**Results:**

The genome sequence of *P. difficile* Z31 is a circular chromosome of 4298,263 bp, with a 29.21 % GC content, encoding 4128 proteins, and containing 78 pseudogenes. This strain belongs to ST 3, clade 1, and has five phage regions in its genome. Genes responsible for resistance to tetracycline and erythromycin were detected and more importantly, Z31 also contains genes that promote spore production and stability, cell attachment, intestinal adherence, and biofilm formation.

**Conclusion:**

In this study, we present the first complete genome sequence of nontoxigenic *P. difficile* strain Z31. When the Z31 genome was compared with those of other isolates available in GenBank, including a draft genome of a nontoxigenic strain, several unique regions were evident. Z31 contains no toxin genes, but encodes several non-toxin virulence factors, which may favor host colonization.

**Electronic supplementary material:**

The online version of this article (doi:10.1186/s13099-016-0095-3) contains supplementary material, which is available to authorized users.

## Background

*Peptoclostridium difficile*, initially called *Bacillus difficilis*, was first isolated from the meconium of newborns by Hall and O’Toole in 1935 [1]. The name ‘*Clostridium difficile*’ was made official in 1980 in the Approved Lists of Bacterial Names [2] based on a phenotypic study by Prevót [3]. Recently, in a study based on 16S rRNA and ribosomal protein sequences, Yutin and Galperin [4] proposed the reallocation of some *Clostridium* species into six new genera, renaming *C. difficile* ‘*Peptoclostridium difficile*’.

**Fig. 1:**
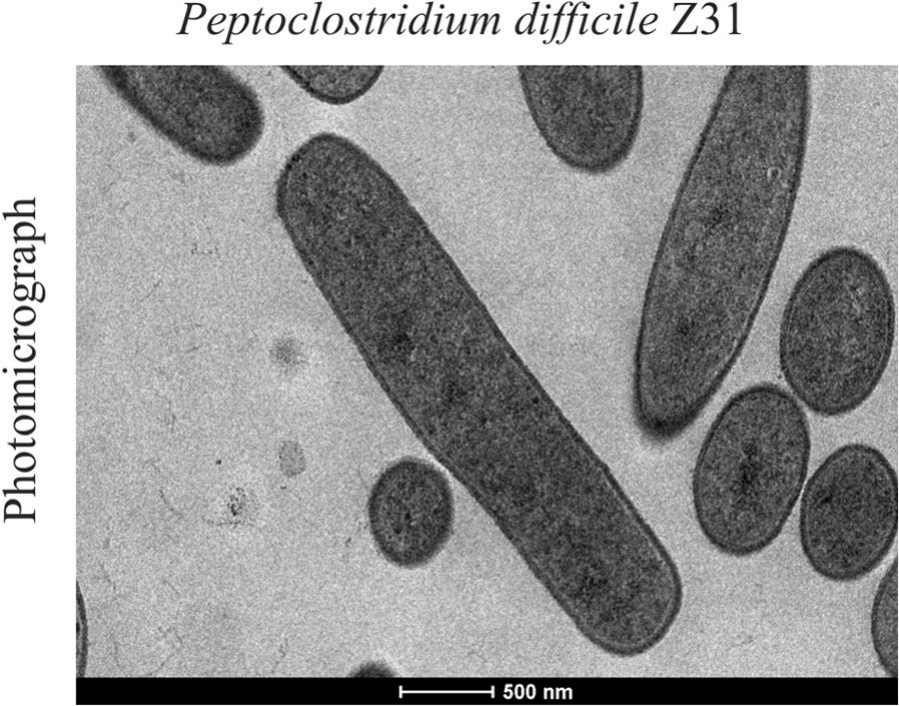
Photomicrograph of *Peptoclostridium difficile* strain Z31

The genus *Peptoclostridium*, in the phylum Firmicutes, class Clostridia, order Clostridiales, and family Peptostreptococcaceae [4], is characterized by strictly anaerobic, motile, pleomorphic Gram-positive bacteria, with dimensions of 0.5–1.9 × 3.0–16.9 µm, which form oval subterminal spores (Fig. 1) with a bacillus cell shape. The bacteria are spore-forming and mesophilic (20–37 °C), with an optimal pH range of neutral to alkaline. They ferment fructose, glucose, levulose, mannitol, mannose, salicin, and usually xylose, but not galactose, glycerol, inulin, lactose, raffinose, or sucrose. They are chemoorganotrophs and can use yeast extract as their sole carbon and energy source and peptone as their nitrogen source. *Peptoclostridium difficile* liquefies gelatin, but does not attack coagulated serum, milk, or meat proteins and is unable to reduce sulfate. It is negative for lecithinase, lipase, oxidase, and catalase. Acetate is produced as a major end product, but it also produces butyrate, formate, isobutyrate, isocaproate, isovalerate, lactate, and valerate [3–5].

Until the late 1970s, *P. difficile* was not recognized as pathogenic bacteria. However, in this decade, *P. difficile* and its toxins were related in fecal contents of human patients with pseudomembranous colitis [6] and the disease was reproduced in hamsters [7], confirming the importance of this microorganism as an enteropathogen. Today, this bacterium is known to be the cause of *P. difficile* infection (PDI), the main cause of nosocomial diarrhea in humans worldwide and a possible cause of diarrhea in general community [8, 9].

In veterinary medicine, *P. difficile* is the most important uncontrolled cause of neonatal diarrhea in piglets in the USA and Europe, and also occurs in other domestic animals and some wild species [10, 11]. In piglets, CDI affects animals to 1–7 days of life, and it was demonstrated that until 1 day of life, 68–100 % of the animals are infected by the microorganism [12, 13]. The disease is subclinical, and just few animals show diarrhea, however, the infection can affect the development of the animals causing economic losses to the farmer [14].

The pathogeny of PDI involves the colonization of colon by some toxigenic strain of *P. difficile* and production of its toxins, the toxin A, an enterotoxin, and toxin B, a cytotoxin, that act synergistically causing cytoskeleton damages, cell rounding, disruption of tight junctions and cell death [15]. The genes responsible to produce toxins, the main difference between toxigenic and nontoxigenic strains, are localized in a pathogenicity locus of 19 kb, called PaLoc [16].

Despite the known importance of *P. difficile* in humans and animals, no vaccine is yet commercially available. Studies have shown that recombinant and classical immunogens expressing toxins A and B can prevent the occurrence of diarrhea or reduce the severity of *P. difficile* infection (PDI) in a rodent model [17]. These vaccines might limit, but cannot prevent, the fecal shedding of the microorganism, which is essential because *P. difficile* is a nosocomial pathogen. Because this bacterium is also a potential zoonotic agent, preventing its colonization of domestic animals should be a priority [10]. Among other alternative preventive strategies examined, the use of nontoxigenic *P. difficile* strains to prevent PDI has been shown to reduce the occurrence of the disease in humans and piglets by preventing their colonization by toxigenic strains [18–21].

There has been no report of the complete genome sequence of a nontoxigenic *P. difficile* strain, a necessary step in understanding this candidate live vaccine. Therefore, in this study, we determined the complete genome sequence of *P. difficile* nontoxigenic strain Z31.

## Methods

### Growth conditions and DNA isolation

*Peptoclostridium difficile* Z31, ribotype 009, a nontoxigenic strain isolated from a healthy dog on February 1, 2009, in the city of Belo Horizonte (state of Minas Gerais, Brazil), was selected for sequencing because it prevented PDI in hamster model [22], similar to some strains previously reported [23]. This strain was grown in Mueller–Hinton agar supplemented with 5 % blood and 0.1 % taurocholate at 37 °C under anaerobic conditions for 48–72 h. Its genomic DNA was extracted with the Maxwell 16^®^ Research Instrument (Promega, USA) combined with lysozyme (10 mg/mL) and proteinase K (20 mg/mL). Briefly, cells were incubated overnight in lysozyme solution (10 mg/mL) at 37 °C. Proteinase K was added and the mixture was incubated at 56 °C for 30 min. According to the kit instructions: (i) the samples were lysed in the presence of a chaotropic agent and a detergent; (ii) the nucleic acids were bound to silica magnetic particles; (iii) the bound particles were washed, to isolate them from other cell components; and (iv) the nucleic acids were eluted into a formulation for sequencing. The extracted DNA was stored at −80 °C until analysis.

### Genome sequencing and assembly

The genome was sequenced with the Ion Torrent PGM™, in a mate-pair sequencing kit with an insert size of 3 kbp (~144-fold coverage) and with a fragment sequencing 400 bp kit (~318-fold coverage). The quality of the raw data was analyzed with FastQC [24] and the sequence was assembled with the Mira 4.9.1 software [25] and Newbler 2.9 (Roche, USA) for the fragment library, and with SPAdes 3.5.0 [26] for the mate-pair library (the parameters for all the assembler software are shown in Additional file 1). This was the ab initio strategy applied to all libraries. The larges contigs obtained with Newbler and Mira were used as input, as trusted-contigs, in SPAdes. We obtained 20 scaffolds, with an N50 value of 698,574 bp, and the largest scaffold had a length size of 1691,449 bp. Gap filling was conducted with CLC Genomics Workbench 7 (Qiagen, USA), after the construction of a super scaffold with the CONTIGuator 2.0 software [27], using the default parameters and *P. difficile* strain CD196 (GenBank: NC_013315.1) as the reference. The gaps in the rRNA operon regions were filled by consensus mapping to the reference, and the remaining gaps were mapped recursively to the raw data on the gap flanks, and it was repeated several times until an overlap was found.

### Genome annotation

The genome was annotated automatically with the Prokka 1.10 software (Rapid Bacterial Genome Annotation) [28], with the default parameters and nested databases in the order: TrEMBL Uniprot containing only (*Pepto*) *Clostridium* spp. proteins and RefSeq database. The genome was also curated manually in all putative frameshifts using the Artemis software [29], based on the coverage visualized with the CLC Genomics Workbench 7 software, with corrected indel assembly bias. Genes encoding signal peptides were identified with the SignalP 4.0 software [30] on a local installation, followed by the identification of transmembrane helices with Tmhmm 2.0 [31] and a Pfam domain search with PfamScan [32]. These three tools were used with their default parameters.

### Multilocus sequence typing (MLST) and in silico PCR

MLST was performed with PubMLST (available at http://pubmlst.org/cdifficile/) using the complete genome sequence. An in silico PCR search for genes related to virulence factors and antimicrobial resistance was performed with the jPCR software [33], with the default parameters and the primer sets shown in Additional file 2.

### Quality assurance

Genomic DNA was isolated from a pure bacterial isolate and confirmed with 16S rRNA gene sequencing. All the raw sequencing data were mapped onto the final genome and the lack of contamination with other genomes was confirmed by the coverage and the low number of unmapped reads.

**Fig. 2:**
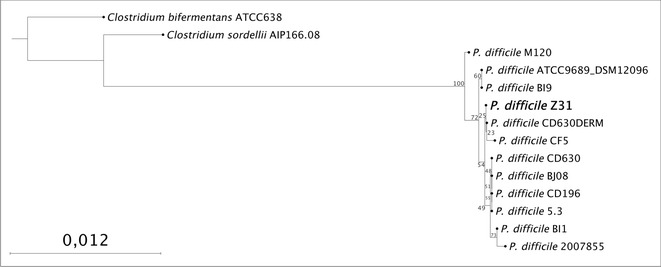
Phylogenetic tree of *Peptoclostridium difficile* strain Z31 representing the relative position in the genus *Peptoclostridium* based on 16S sequences. The statistical method used was maximum likelihood and the bootstrap number was 1000. Thus, the values next to the nodes represent the percentage on the number of times, in 1000 repetitions, in which that clade was formed

An alignment was constructed with the 16S rRNA sequence regions on the assembled scaffolds, predicted with the Barrnap software (available at https://github.com/tseemann/barrnap), and the 16S rRNA genes of genomes available in GenBank. A phylogenetic tree was constructed from this alignment with the neighbor-joining method based on 1000 randomly selected bootstrap replicates, using the CLC Genomic Workbench 7.0 software. On the tree, strain Z31 was positioned among other *P. difficile* strains (Fig. 2).

## Results and discussion

After the genome assembly, gap filling, and annotation process, an in silico PCR was performed through searching for genes related to virulence factor, antibiotic resistance, and other known toxins. Considering the perspective of using the nontoxigenic strain Z31 to prevent PDI by competitive exclusion, some non-toxin virulence factors are desirable, predominantly those factors responsible for spore production and stability and those that promote cell attachment and host colonization. Z31 is positive for Cwp84 and surface-layer protein A (SlpA). SlpA is considered the major factor responsible for bacterial intestinal adherence, and Cwp84 is essential for the formation of that protein [34, 35]. GroEL, Cwp66, and a fibronectin-binding protein (Fbp68), which are also important in host-cell adherence, were also found [34–38]. Strain Z31 was also positive for genes encoding the flagellar proteins FliC and FliD, which play roles in the colonization and adherence of Z31 in vivo and are essential in later stages of biofilm formation [39–41]. These factors found in Z31 related to cell attachment are extremely important, because non toxigenic strains have to be able to compete with toxigenic strains by the colonization sites to prevent the disease [23].

The gene encoding the major regulator of sporulation in *P. difficile*, *Spo0A*, was detected in this strain. An absence or deficiency of Spo0A can cripple or impair the sporulation process [35, 42]. Genes encoding five spore coat proteins (*cotA*, *cotB*, *cotC*, *cotD*, and *sodA*) were also detected. The cotA protein is the most important protein in stabilizing the spore coat and ensures the integrity of this structure [43]. The formation of stable spores is also important for a nontoxigenic strain candidate to prevent the disease, because the bacteria need to pass through the stomach and be able to colonize the colon [23]. Vegetative cells are sensible to low pH, on the other hand, the spores resist to this conditions, allowing a great number of viable particles reaches the colon [44]. Genes responsible for resistance to tetracycline (*tetM*) and erythromycin (*ermG*) were also detected with previously described PCR primers [45, 46]. In contrast, none of the genes encoding proteins directly linked to toxin production were detected (*tcdA*, *tcdB*, *tcdC*, *cdtA*, or *cdtB*) [47] confirming the absence of the pathogenicity locus (PaLoc), which is essential for *P. difficile* infection [48].

Furthermore, the complete genomes of this species available at GenBank were selected to perform a similarity analysis with Gegenees software [49] with sequence fragmentation length of 500 nucleotides and a threshold of 30 %. Also, two complete genomes of species of the *Clostridium* genus were included as an outgroup. The similarity matrix was used to generate a heatplot and a “.nexus” format for phylogenomic analysis (Additional file 3). Although the Z31 strain is a nontoxigenic strain, the Additional file 3 shows that clade of this strain is paraphyletic with the type strain ATCC9689, a known as toxigenic strain, suggesting an evolutionary derivation of a same organism. Thereby, the nontoxigenic behavior of the Z31 strain seems to be occasioned by the losses of the toxin genes.

### Initial findings

**Fig. 3:**
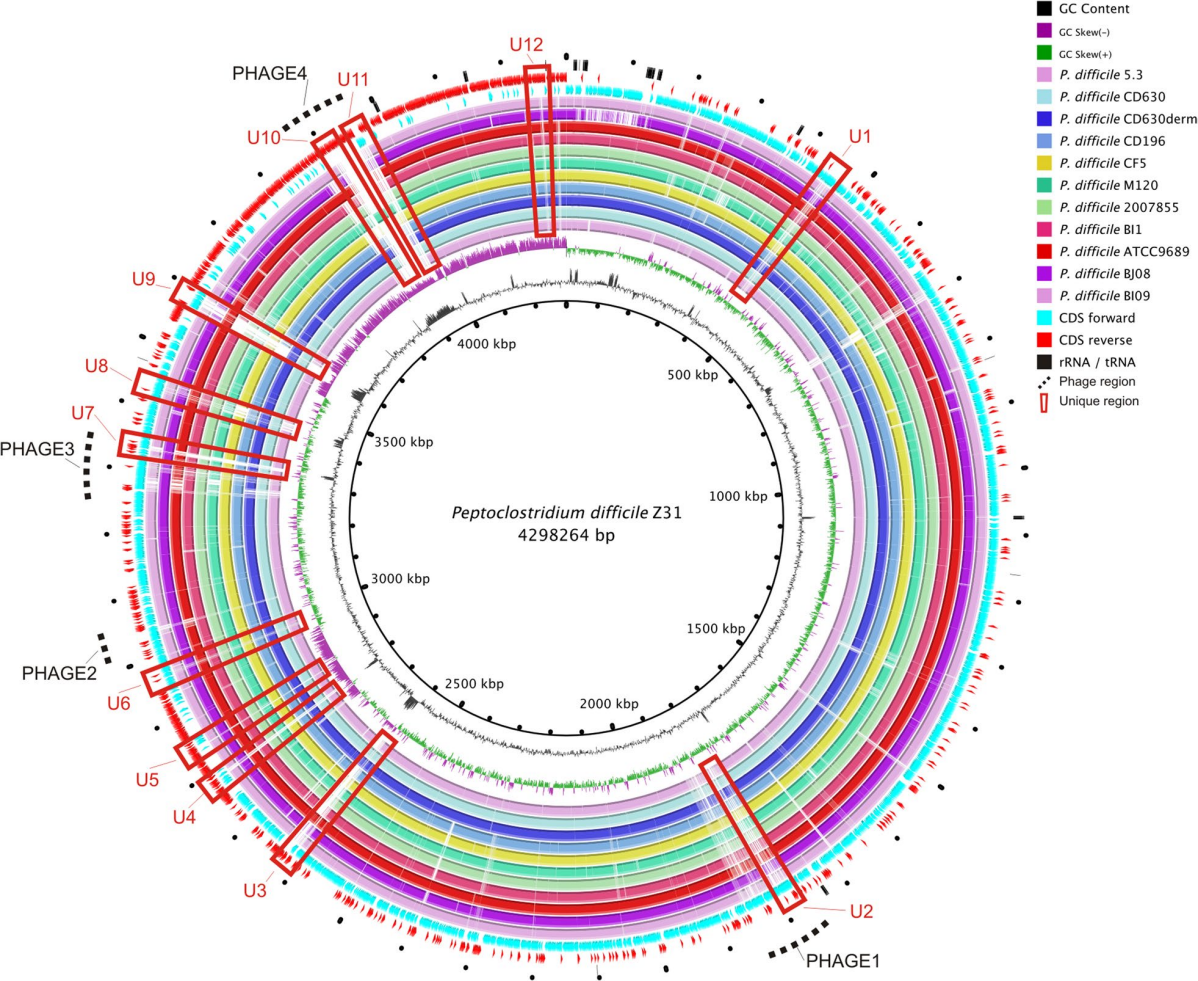
Graphical circular map of *Peptoclostridium difficile* strain Z31 genome. From outside to the center: predicted phage regions by PHAST; RNAs; CDSs on reverse strand; CDSs on forward strand; Blastn hits with BI9, BJ08, ATCC9689/DSM1296, BI1, 2007855, M120, CF5, CD196, CD630DERM, CD630, Cd5.3 strains; GC skew; and, GC content

The *P. difficile* genome is composed of a circular chromosome of 4298,263 bp. The GC content is 29.21 % and the genome contains 78 pseudogenes. Briefly, the genome has 4206 CDSs, and encodes 29 rRNAs, one transfer–messenger RNA (tmRNA), and 58 tRNAs. Table 1 summarizes the subset of the 3809 genes with predicted functions that are associated with each COG functional categories. In summary, 3324 genes were predicted to have Pfam domains, 166 to have signal peptides, and 1011 to have transmembrane helices. No CRISPR repeats were found. Figure 3 shows the disposition of RNAs and CDSs coding sequences on the forward and reverse strands, the GC content, and the GC skew.

**Table 1:** Number of genes associated with general COG functional categories [55]

Code	Value^b^	%age^a^	Description
J	238	5.6585	Translation, ribosomal structure and biogenesis
A	0	0	RNA processing and modification
K	479	11.3884	Transcription
L	184	4.3747	Replication, recombination and repair
B	1	0.0237	Chromatin structure and dynamics
D	68	1.6167	Cell cycle control, cell division, chromosome partitioning
V	134	3.1859	Defense mechanisms
T	327	7.7746	Signal transduction mechanisms
M	208	4.9453	Cell wall/membrane biogenesis
N	79	1.8782	Cell motility
U	40	0.9510	Intracellular trafficking and secretion
O	111	2.6390	Posttranslational modification, protein turnover, chaperones
C	219	5.2068	Energy production and conversion
G	310	7.3704	Carbohydrate transport and metabolism
E	321	7.6319	Amino acid transport and metabolism
F	93	2.2111	Nucleotide transport and metabolism
H	147	3.4950	Coenzyme transport and metabolism
I	89	2.1160	Lipid transport and metabolism
P	168	3.9942	Inorganic ion transport and metabolism
Q	57	1.3552	Secondary metabolites biosynthesis, transport and catabolism
R	345	8.2025	General function prediction only
S	251	5.9676	Function unknown
–	2	0.0475	Not in COGs

^a^The percentage is based on the total number of protein coding genes in the annotated genome

^b^The total not correspond to the final quantity of CDSs for each genome, because some genes are associated with more than one COG functional categories

When the genome of Z31 was compared with those of other *P. difficile* strains deposited in GenBank [50], it showed high similarity to them (95.50 ± 2.68 %—Additional file 3), with the exception of some genomic islands (Fig. 3), four of which were predicted with PHAST [51] to be phage regions. A brief description of these phages is given in Table 2.

**Table 2:** Phage summary predicted by PHAST

Number	Length (kbp)	Completeness	First common name	Keyword	GC content
Phage 1	96.3	Intact	Clostr_phi_CD119	Integrase, terminase, portal, head, capsid, tail, lysin, plate, and protease	28.9
Phage 2	24.1	Incomplete	Clostr_phi_CD119	Tail, lysin, and plate	27.9
Phage 3	63.6	Intact	Clostr_phiC2	Integrase, terminase, portal, head, capsid, tail, and lysin	28.6
Phage 4	138.1	Intact	Bacill_G	Protease, recombinase, tail, transposase, integrase, head, capsid, portal, and terminase	35.7

A robust high-throughput MLST scheme for *P. difficile* was developed and validated [52], and allowed this species to be genotyped directly. Z31 was typed with MLST at loci *adk 1*, *atpA 1*, *dxr 2*, *glyA 1*, *recA 1*, *sodA 1*, and *tpi 1*, which classified this strain as ST3 in MLST clade 1. This result corroborates previous work, which reported that strains from PCR ribotype 009 are commonly classified as ST3 [53]. Strain Z31, *P. difficile* ATCC9689/DSM1296, and *P. difficile* BI9 were the only three ST3 strains identified among the strains whose complete genomes or near-complete genomes (e.g., one scaffold) are deposited in GenBank. However, Z31 contain some unique regions, as shown in Fig. 3 (U1–U12). In contrast, *P. difficile* 5.3, described as nontoxigenic by Darling et al. [54], belongs to ST15, clade 1, a common classification for strains of PCR ribotype 010.

### Future directions

Further analysis of the *P. difficile* Z31 genome will provide new information about the adaptation of this strain to the gastrointestinal tract, and new insights into its inhibition of toxigenic *P. difficile* strains.

### Availability of supporting data

This whole-genome shotgun sequence has been deposited in the DDBJ/EMBL/GenBank databases under accession number CP013196. The version described in this paper is the first version CP013196.1.

## Additional files

**Additional file 1.** Assembler software (Mira, Newbler and SPAdes) parameters.

**Additional file 2.** Primer sets used on in silico PCR for genes related to virulence factor, antibiotic resistance, and other known toxins.

**Additional file 3.** Phylogenomic analysis performed using Gegenees.

## Abbreviations

GC content: number of G and C nucleotides
rRNA: ribosomal RNA
PDI: *Peptoclostridium difficile* infection
PGM: Personal Genome Machine
tmRNA: transport–messenger RNA
tRNA: transport RNA
MLST: multilocus sequence typing
