# Supplementary figures and images for: Complete genome sequence of Peptoclostridium difficile strain Z31
Source: Gut Pathog. 2016 Apr 1;8:11. doi: 10.1186/s13099-016-0095-3 (PMC5562067; doi:10.1186/s13099-016-0095-3)

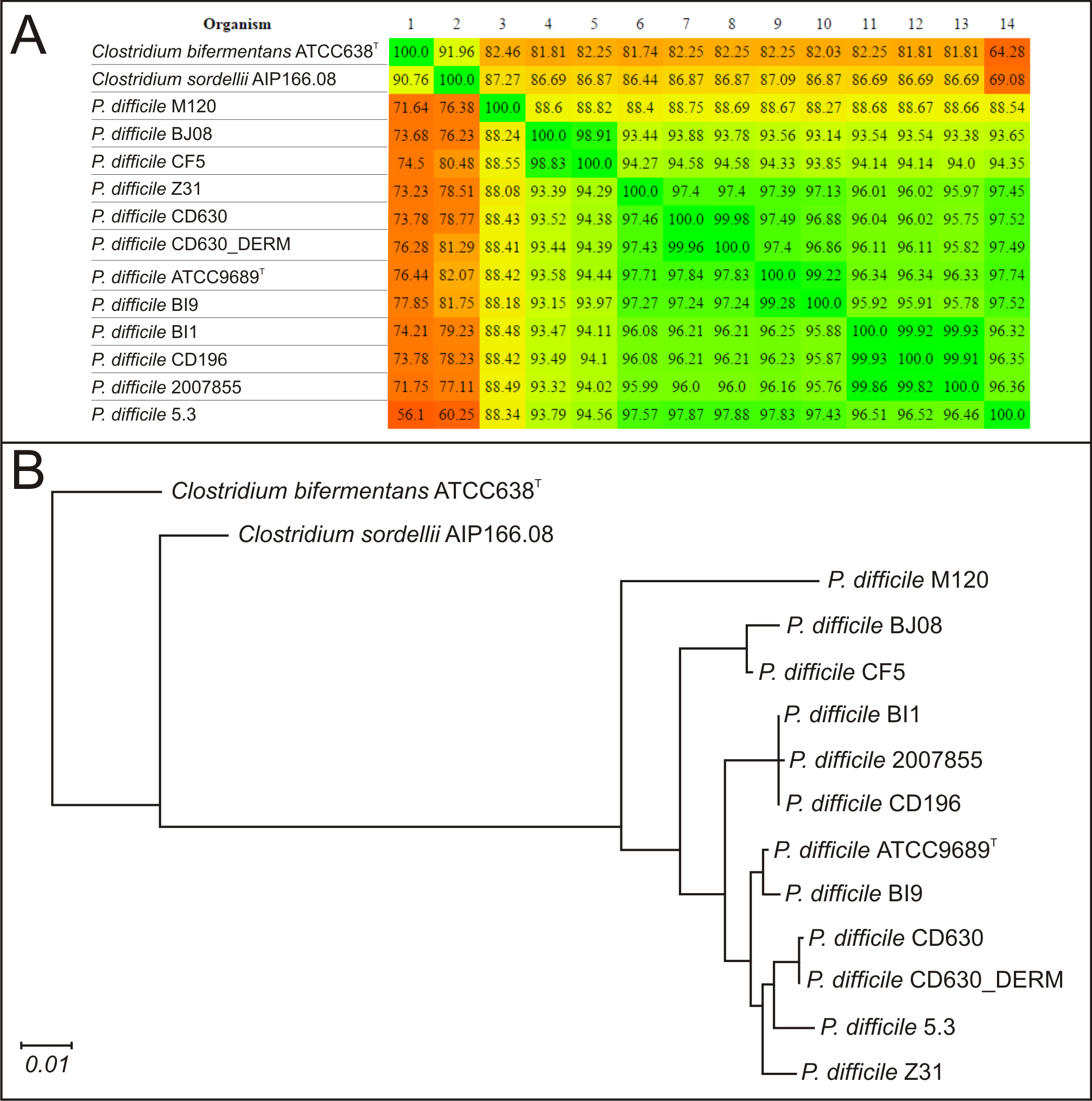

Supplement: Supplementary file 3 — Additional file 3. Phylogenomic analysis performed using Gegenees. [file 13099_2016_95_MOESM3_ESM.tif]
